# Integrating Host Phylogeny and Bacterial Detection Patterns Reveals Contrasting Associations of *Buchnera aphidicola* and Other Aphid-Associated Bacteria

**DOI:** 10.3390/insects17080875

**Published:** 2026-08-21

**Authors:** Işıl Özdemir, Naciye Sena Çağatay, Nurper Guz

**Affiliations:** 1 Department of Plant Protection, Faculty of Agriculture, Kocaeli University, 41285 Kocaeli, Türkiye; 2 Acarology Laboratory, Department of Plant Protection, Agricultural Faculty, Cukurova University, 01330 Adana, Türkiye; 3 Institute of Infection, Veterinary and Ecological Sciences, University of Liverpool, Liverpool L69 7ZX, UK; 4 Gümüşdere Yerleşkesi Keçiören, Biotechnology Institute, Ankara University, 06100 Ankara, Türkiye; nurperguz@agri.ankara.edu.tr

**Keywords:** aphid–bacteria interactions, *Buchnera aphidicola*, aphid-associated bacteria, host phylogeny, cophylogenetic analysis, diagnostic PCR

## Abstract

Aphids are important agricultural pests that interact with diverse bacterial taxa living within or associated with their bodies. Some bacteria, such as the obligate symbiont *Buchnera aphidicola*, provide essential nutritional functions and are predominantly inherited vertically, whereas other aphid-associated bacteria may have more variable distributions and ecological roles. In this study, we investigated patterns of association between aphid evolutionary relationships and selected bacterial taxa. Aphids were identified using morphological characteristics and mitochondrial cytochrome c oxidase subunit I (COI) sequences, and host phylogenetic relationships were reconstructed using mitochondrial DNA data. We then compared the host phylogeny with that of the obligate symbiont *Buchnera aphidicola* based on 16S rRNA sequences and screened aphid specimens for selected aphid-associated bacteria (*Wolbachia*, *Pantoea*, and *Arsenophonus*) using diagnostic PCR assays. The host and *Buchnera* phylogenies showed significant global congruence, consistent with a strong long-term host-associated evolutionary relationship and predominantly vertical inheritance of *Buchnera*. In contrast, the selected aphid-associated bacteria showed heterogeneous detection patterns across the sampled aphid taxa. *Pantoea* was detected relatively frequently and across a broad range of sampled hosts, whereas *Wolbachia* and *Arsenophonus* showed more variable and restricted detection patterns. Given the small and uneven sample sizes among aphid species, these findings are interpreted as descriptive screening patterns rather than species-level prevalence estimates. Overall, the results highlight contrasting patterns of host-associated evolution between *Buchnera* and other aphid-associated bacteria and provide a basis for further investigation of the ecological and evolutionary processes underlying these associations.

## Simple Summary

Aphids are important agricultural pests that can damage crops and transmit plant diseases. Their survival and adaptation are closely associated with bacteria that live within or on their bodies. Some bacteria are essential for aphid nutrition and survival, whereas others may influence their ability to cope with environmental stress or interact with other organisms. This study investigated whether bacteria associated with aphids show evolutionary patterns related to those of their hosts. We identified aphid species using morphological characteristics and genetic information and compared their evolutionary relationships with those of their obligate bacterial symbiont, *Buchnera aphidicola*. We also screened aphid specimens for three other bacterial taxa, *Wolbachia*, *Pantoea*, and *Arsenophonus*. Aphid and *Buchnera* phylogenies showed closely matching evolutionary patterns, supporting their long-term association and predominantly vertical transmission from mother to offspring. In contrast, the other bacteria showed more variable distributions among aphid species. *Pantoea* was detected across a relatively broad range of sampled aphids, whereas *Wolbachia* and *Arsenophonus* were detected less consistently. These findings demonstrate that different bacteria associated with aphids can exhibit distinct evolutionary patterns and provide useful insights into aphid–bacteria interactions.

## 1. Introduction

Aphids (Hemiptera: Aphididae) are among the most economically significant agricultural pests worldwide, causing substantial crop losses through direct phloem feeding and the transmission of numerous plant [1,2,3,4,5]. A key factor underlying their ecological success is their association with bacterial symbionts, which play central roles in nutrition, stress tolerance, and ecological adaptation [6,7,8].

All aphids harbor the obligate endosymbiont *Buchnera aphidicola*, which provides essential amino acids lacking in plant phloem sap, thereby enabling host survival and reproduction [6,9]. This mutualistic association is characterized by strict vertical transmission and long-term evolutionary stability, resulting in strong phylogenetic congruence between aphids and *Buchnera* lineages [10,11]. Over evolutionary time, this relationship has led to extensive genome reduction in *Buchnera*, reflecting its specialization as an intracellular symbiont [9,12]. Variation among *Buchnera* strains has been shown to influence host adaptation, including thermal tolerance and host plant specialization [9,10,11,12,13].

In addition to *Buchnera*, aphids commonly harbor facultative symbionts such as *Hamiltonella defensa*, *Serratia symbiotica*, and *Arsenophonus*, which can confer ecologically relevant benefits including parasitoid resistance and thermal tolerance [14,15]. Other bacteria, including *Wolbachia* and environmental taxa such as *Pantoea*, have also been detected in aphids; however, their functional roles appear to be more context-dependent and remain less well resolved [15,16,17]. Facultative symbionts are known to meditate a wide range of phenotypic effects, including resistance to natural enemies, tolerance to environmental stress, and expansion of host plant range [18,19,20]. Previous studies suggest that facultative symbionts may contribute to nutritional complementation under variable environmental conditions [14,21].

Despite extensive characterization of aphid symbionts, it remains unresolved whether obligate and facultative symbionts are structured by similar phylogenetic constraints or reflect fundamentally different ecological processes [9,14]. Resolving this distinction is critical for understanding how symbiont diversity contributes to host adaptation and evolutionary trajectories. Unlike the obligate symbiont *Buchnera*, facultative symbionts exhibit more dynamic transmission modes, including horizontal transfer between host lineages, resulting in highly variable and often heterogeneous distribution patterns across aphid species and populations [14,20]. This contrast points to fundamentally different evolutionary patterns, with obligate symbionts tightly linked to host lineage history and facultative taxa shaped by ecological context, horizontal transmission, and selective pressures.

Characterizing how these symbionts are distributed across hosts and how they co-occur remains essential for elucidating their ecological roles and evolutionary dynamics. Yet, it remains unclear whether symbiont distribution patterns are shaped by host phylogeny or by ecological factors. This uncertainty is particularly evident when obligate and facultative symbionts are examined within a unified framework. Previous research has demonstrated that symbiont associations within hosts may be structured, with both synergistic and antagonistic interactions shaping symbiont community structure [22,23]. However, detecting such patterns remains challenging due to limited sample sizes; uneven species representation; and the complex interplay between host phylogeny, symbiont transmission, and environmental factors.

Although aphid–symbiont systems have been widely studied, integrative approaches remain relatively limited. Few studies have combined host phylogeny, obligate symbiont phylogeny, and quantitative analyses of facultative symbiont prevalence across multiple species, especially at regional scales [14,24]. In many systems, the relationship between phylogenetic structure and symbiont distribution has not been explicitly evaluated within a unified analytical framework [9,14].

Here, we integrate host phylogenetic reconstruction with targeted symbiont screening to investigate how different components of the aphid-associated bacterial community are distributed across host lineages. Specifically, we aimed to (i) reconstruct aphid phylogenetic relationships using mitochondrial COI sequences; (ii) examine phylogenetic patterns of the obligate symbiont *Buchnera aphidicola* based on 16S rRNA sequences; and (iii) characterize the distribution and prevalence of selected symbiont taxa across aphid hosts within a phylogenetic framework.

## 2. Materials and Methods

### 2.1. Sample Collection and Species Identification

Aphid specimens were collected from multiple host plant species across different locations in Ankara/Türkiye (Table 1) between May and August 2017. Sampling was carried out in regions where fruit cultivation areas are dense. Individual samples were preserved in 96% ethanol immediately after collection and stored at −20 °C prior to molecular analyses. Species identification was initially performed based on morphological diagnostic characteristics under a stereomicroscope [1,5,25] and subsequently validated using *mitochondrial cytochrome oxidase I* (*COI*) barcoding to confirm species identity. A total of 40 individual aphid specimens representing multiple species were included in downstream analyses.

### 2.2. DNA Extraction, PCR Amplification, and Sequencing

Genomic DNA was isolated from individual aphid specimens using the High Pure PCR Template Preparation Kit (Roche, Mannheim, Germany) following the manufacturer’s instructions with minor modifications to improve DNA yield from small-bodied specimens. DNA integrity was verified by electrophoresis on 1% agarose gels, and concentration and purity were assessed using a Nanodrop 2000 spectrophotometer (Thermo Scientific, Wilmington, DE, USA). A 658 bp fragment of the mitochondrial *cytochrome c oxidase subunit I* (*COI*) gene was amplified using the universal primer pair LCO1490 and HCO2198 [26]. PCR reactions were performed in a final volume of 50 µL containing 1 µL of DNA template, 1× GoTaq Flexi reaction buffer (Promega Corporation, Madison, WI, USA), 2.5 mM MgCl_2_, 0.2 mM of each dNTP, 0.5 µM of each primer, and 1.25 U of GoTaq DNA polymerase. Nuclease-free water was added to obtain a final volume of 50 µL. The thermal cycling profile consisted of initial denaturation at 94 °C for 2 min and 35 cycles of 94 °C for 30 s, 50 °C for 45 s, and 72 °C for 2 min, followed by final extension at 72 °C for 10 min.

In addition to host COI amplification, sequence characterization of the obligate endosymbiont *Buchnera aphidicola* was amplified by a fragment of the bacterial 16S rRNA gene. Multiple *B. aphidicola* 16S rRNA sequences representing diverse aphid-associated lineages were retrieved from the NCBI database and aligned using MAFFT [27] to identify conserved regions suitable for primer design. Since this study aimed to investigate whether *Buchnera* phylogenetic patterns correspond with those of different aphid lineages, a new primer pair was designed to enable consistent amplification across multiple aphid taxa included in this study. Primer design was performed using Primer 3 based on conserved regions of the aligned sequences, with parameters including a melting temperature (Tm) range of 55–60 °C and GC content between 40 and 60%.

The designed primer pair BA-F (5′-AGCGTTAATCAGAATTACTGGG-3′) and BA-R (5′-GTTTATAACCGGCAGTCTCC-3′) amplifies an approximately 700 bp fragment of the *Buchnera* 16S rRNA gene. Primer specificity was evaluated in silico using Primer-BLAST (National Center for Biotechnology Information, Bethesda, MD, USA)against the NCBI database, confirming specific matches with *B. aphidicola* sequences. PCR reactions were performed under conditions similar to those described above, with minor adjustments to the annealing temperature when required.

All PCR amplicons (*COI* and *Buchnera 16S rRNA*) were visualized on 2% agarose gels and purified using the Wizard SV Gel and PCR Clean-Up System (Promega). Bidirectional sequencing was performed using the DTCS Quick Start Kit (Beckman Coulter). Sequencing reactions were purified with the Agencourt CleanSeq system (Agencourt Bioscience Corporation, Beverly, MA, USA) and analyzed on a GenomeLab GeXP Genetic Analysis System (Beckman Coulter, Inc., Fullerton, CA, USA). Chromatograms were assembled, inspected, and edited manually in Geneious R9 (Biomatters Ltd., Auckland, New Zealand), and consensus sequences were aligned using MAFFT v7 [27].

### 2.3. Sequence Processing and Phylogenetic Analyses

Forward and reverse chromatograms were manually inspected and edited in Geneious R9. Consensus sequences were aligned using MAFFT v7 [27], and poorly aligned or ambiguous regions were removed using TrimAl version 1.5.1 (Barcelona, Spain) [28]. The final datasets consisted of 57 COI sequences (402 bp) and 52 *Buchnera aphidicola* 16S rRNA sequences (577 bp), including reference sequences retrieved from GenBank (NCBI). Phylogenetic analyses were conducted using IQ-TREE v1.6.12 [29] under a maximum likelihood framework. The best-fit nucleotide substitution models were selected using ModelFinder version 3.1.3 [30] based on the Bayesian Information Criterion (BIC), with TIM2+F+I+G4 selected for the COI dataset and TPM3u+F+I selected for the 16S dataset. Branch support was assessed using ultrafast bootstrap approximation (1000 replicates) and SH-aLRT tests (1000 replicates). The sequence of *Adelges tsugae* (KR0372787.1), retrieved from GenBank, was used as the outgroup to root the COI-based aphid phylogeny, whereas the sequence of *Escherichia coli* (J01859.1) was used as the outgroup to root the 16S rRNA-based *Buchnera aphidicola* phylogeny. The mitochondrial COI sequences generated from the aphid specimens were deposited in GenBank under accession numbers PZ830616-PZ830655, while the 16S rRNA sequences generated from *Buchnera* specimens were deposited in GenBank under accession numbers PZ836554-PZ836592. The GenBank accession numbers are provided in Table 1.

### 2.4. Cophylogenetic Analysis

Cophylogenetic analyses were performed using the maximum likelihood (ML) phylogenetic trees reconstructed for aphid hosts based on mitochondrial COI sequences and their corresponding *Buchnera aphidicola* 16S rRNA sequences. ML trees generated in IQ-TREE were imported into R version 4.5.2 [31] for subsequent analyses. The R packages ape v5.8-1 [32], phytools v2.5-2 [33], and paco v0.5-0 [34] were used for phylogenetic data manipulation, visualization, and cophylogenetic analyses.

Prior to statistical analyses, outgroup taxa were removed from both host and symbiont phylogenies to prevent their influence on the estimation of host–symbiont congruence. Outgroups were retained only for graphical representation to preserve the original rooting of the phylogenetic trees. A one-to-one host–symbiont association matrix was constructed by matching each aphid specimen with the corresponding *B. aphidicola* sequence obtained from the same individual.

Global cophylogenetic congruence between host and symbiont phylogenies was evaluated using the Procrustean Approach to Cophylogeny (PACo) implemented in the paco package in R. Patristic distance matrices derived from the ML phylogenies were calculated using ape, and principal coordinate analysis with Cailliez correction was applied before Procrustes superimposition. Statistical significance was assessed using 9999 permutations. Individual host–symbiont associations were further evaluated using jackknife residual values.

For visualization, host and symbiont phylogenies were displayed as tanglegrams using the cophylo function implemented in the phytools package. The final figure was refined using Adobe Illustrator 30.6 (Adobe Inc., San Jose, CA, USA).

### 2.5. Detection of Aphid-Associated Bacteria

The detection of selected aphid-associated bacteria (*Wolbachia*, *Pantoea*, and *Arsenophonus*) was assessed in 33 aphid specimens using diagnostic PCR assays with the taxon-specific primers and annealing temperatures listed in Table 2, following the general PCR conditions described above. Although 40 aphid specimens were included in the COI-based identification and phylogenetic analyses, seven specimens were not included in the bacterial screening: *Aphis pomi* specimens 52 and 53, *Myzus persicae* specimens 54 and 62, *Myzus cerasi* specimen 55, *Aphis spiraecola* specimen 57, and *Hyalopterus amygdali* specimen 60. These seven specimens were therefore excluded from the bacterial screening dataset.

The bacterial taxa were selected because diagnostic PCR primers for their detection were already available in our laboratory and had been successfully used in previous studies. Therefore, the screening was designed as a targeted assessment of selected aphid-associated bacteria rather than a comprehensive survey of the aphid-associated bacterial community. These assays were used to determine the detection status of each bacterial taxon based on PCR amplification. Each reaction included positive and negative controls to verify amplification specificity and minimize the possibility of contamination. Detection results were recorded as binary data (1 = detected; 0 = not detected) for each individual aphid specimen included in the bacterial-screening analysis.

### 2.6. Detection and Distribution Patterns of Aphid-Associated Bacteria

The detection frequency of aphid-associated bacteria was calculated as the proportion of PCR-positive individuals relative to the total number of 33 aphid specimens included in the bacterial-screening analysis. Given the small and uneven sample sizes across aphid species, the results were interpreted as descriptive screening patterns rather than species-level prevalence estimates. Exact 95% binomial confidence intervals were calculated using the Clopper–Pearson method to describe the uncertainty associated with the observed detection frequencies. Overall detection frequencies were visualized using bar plots with error bars, whereas species-level detection patterns were presented using point-range plots with sample sizes (*n*) indicated for each species. Species represented by small sample sizes (*n* ≤ 3) were visually highlighted to indicate greater uncertainty, and no direct comparisons were made among these groups. To illustrate the distribution patterns of detected bacterial taxa across aphid hosts, a heatmap of raw detection frequencies (%) was generated without clustering, with species ordered taxonomically to facilitate biological interpretation.

### 2.7. Association Analyses Among Detected Aphid-Associated Bacteria

Pairwise associations among detected aphid-associated bacteria (*Wolbachia*, *Pantoea*, and *Arsenophonus*) were evaluated using Fisher’s exact test because of the sparse contingency data from 33 aphid specimens included in the bacterial screening analysis. For each bacterial taxon pair, 2 × 2 contingency tables were constructed, and odds ratios (ORs) were calculated to quantify the direction and strength of co-detection patterns. When zero counts occurred, a Haldane–Anscombe correction of 0.5 was applied. Confidence intervals for ORs were estimated on the log scale and backtransformed to the original scale. Results were visualized using forest plots on a logarithmic scale, with OR = 1 representing the null expectation. Statistical significance was assessed at α = 0.05, and *p*-values were reported for each comparison.

## 3. Results

### 3.1. Aphid Species Composition and Host Plant Associations

A total of 40 aphid specimens representing multiple species and host plant associations were included in the analysis (Table 1). The sampled individuals comprised a taxonomically diverse set of aphid taxa collected from various host plants, with most specimens associated with *Prunus*, *Malus*, and *Pyrus*, and additional records from *Juglans*, *Cydonia*, *Punica*, *Corylus*, and *Populus*. Several aphid species were represented by multiple individuals, including *Aphis gossypii* Glover, *Hyalopterus amygdali* (Blanchard), *Aphis spiraecola* Patch, and *Hyalopterus arundiniformis* Ghulamullah, whereas other taxa were represented by limited sample sizes (*n* ≤ 3).

Host plant associations showed apparent patterns among aphid genera. *Hyalopterus* species were predominantly associated with *Prunus* hosts, with occasional records from secondary hosts such as *Populus*, whereas Aphis species were recorded from multiple plant genera. In contrast, some taxa showed more restricted host associations, including *Panaphis juglandis* (Goeze) associated with *Juglans regia* L. and *Myzocallis coryli* (Goeze) associated with *Corylus avellana* (Table 1).

### 3.2. Molecular Identification and Phylogenetic Analysis

**Figure 1:**
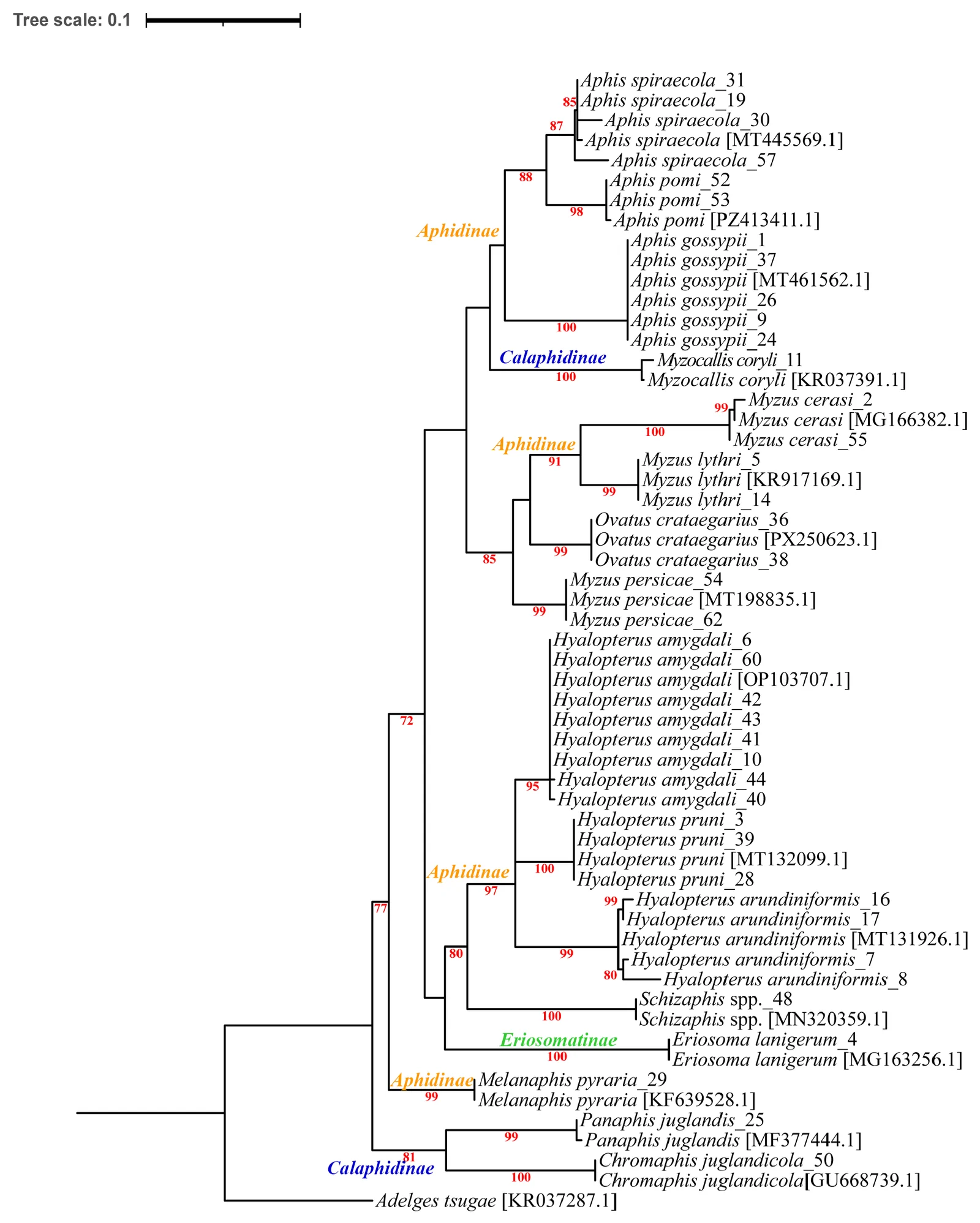
Maximum likelihood phylogenetic tree based on mitochondrial *COI* sequences. Phylogenetic relationships among aphid species were inferred using maximum likelihood analysis of *COI* sequences. Newly generated sequences are shown alongside reference sequences obtained from GenBank (accession numbers indicated). Bootstrap support values (%) are provided at nodes, with only values ≥70% displayed. *Adelges tsugae* (KR0372787.1) was used as an outgroup. Branch lengths correspond to nucleotide substitutions per site, and the scale bar indicates evolutionary distance.

Phylogenetic relationships among the analyzed aphid specimens were reconstructed based on mitochondrial COI sequences (Figure 1). The maximum likelihood (ML) tree showed clear clustering of individuals according to their morphological species identifications. Specimens assigned to the same species based on morphological characteristics generally grouped into distinct clades, with moderate to high bootstrap support values, particularly at terminal nodes (bootstrap support ranging from 72 to 100). Major aphid lineages, including *Hyalopterus*, *Aphis*, and *Myzus*, formed distinct clusters corresponding to their expected taxonomic relationships. Within the *Hyalopterus* lineage, specimens morphologically identified as *H. amygdali* (Blanchard), *H. pruni* (Geoffroy), and *H. arundiniformis* Ghulamullah were resolved into closely related but distinguishable subclades. Similarly, morphologically identified *Aphis* species, including *A. gossypii* Glover, *A. spiraecola* Patch, and *A. pomi* De Geer, formed coherent clusters, whereas *Myzus* species were recovered as a separate lineage. Representative species of the subfamilies Aphidinae, Calaphidinae, and Eriosomatinae recovered across distinct clades reflecting their taxonomic placements. Reference sequences obtained from public databases clustered with the corresponding study specimens, further supporting the consistency between morphological identification and COI-based phylogenetic placement. The phylogeny was rooted using *Adelges tsugae* (KR037287.1) as the outgroup (Figure 1).

### 3.3. Buchnera aphidicola Phylogenetic Analysis

**Figure 2:**
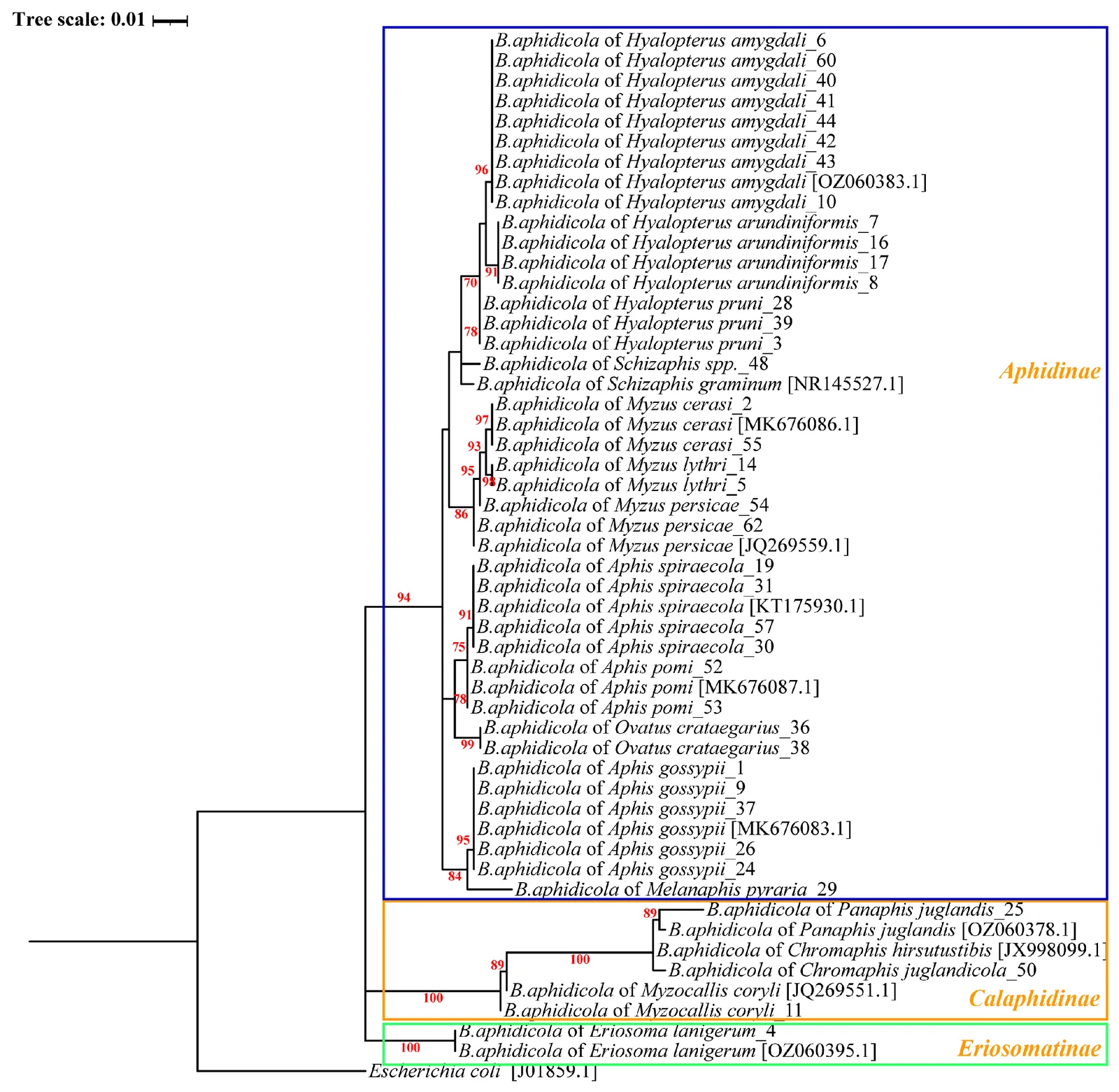
Maximum likelihood phylogenetic reconstruction of *Buchnera aphidicola* based on 16S rRNA sequences. *Buchnera* sequences generated from aphid samples were analyzed together with reference sequences from GenBank (accession numbers provided). Sequences are labeled according to host species and sample identifiers. Bootstrap support values (%) are shown at nodes (only ≥70% displayed). Branch lengths correspond to substitutions per site, as indicated by the scale bar. The resulting topology shows broad clustering of *Buchnera* sequences by host-associated groups. Because the inference is based on partial 16S rRNA data, the tree should be interpreted as a low-resolution view of symbiont phylogenetic structure.

Phylogenetic relationships of the obligate endosymbiont *Buchnera aphidicola* were reconstructed based on partial 16S rRNA gene sequences (Figure 2). The maximum likelihood tree showed a general pattern of clustering among *Buchnera* sequences according to their aphid host lineages, recovering three distinct major clades corresponding to the subfamilies Aphidinae, Calaphidinae, and Eriosomatinae. Sequences associated with *Hyalopterus* species, including *H. amygdali* and *H. pruni*, formed distinct clusters, whereas *Buchnera* sequences associated with *Aphis*, *Myzus*, and related aphid taxa grouped with corresponding reference sequences retrieved from public databases within the Aphidinae clade. Most terminal clades received moderate to high bootstrap support (bootstrap values ranging from 70 to 100), whereas deeper internal nodes showed comparatively lower support. Although the overall topology indicated host-associated structuring of *Buchnera* lineages reflecting major host subfamilies, some localized inconsistencies were observed among specific host–symbiont associations. The phylogeny was rooted using *Escherichia coli* (J01859.1) as the outgroup (Figure 2).

### 3.4. Cophylogenetic Relationship Between Aphid Hosts and Buchnera aphidicola

**Figure 3:**
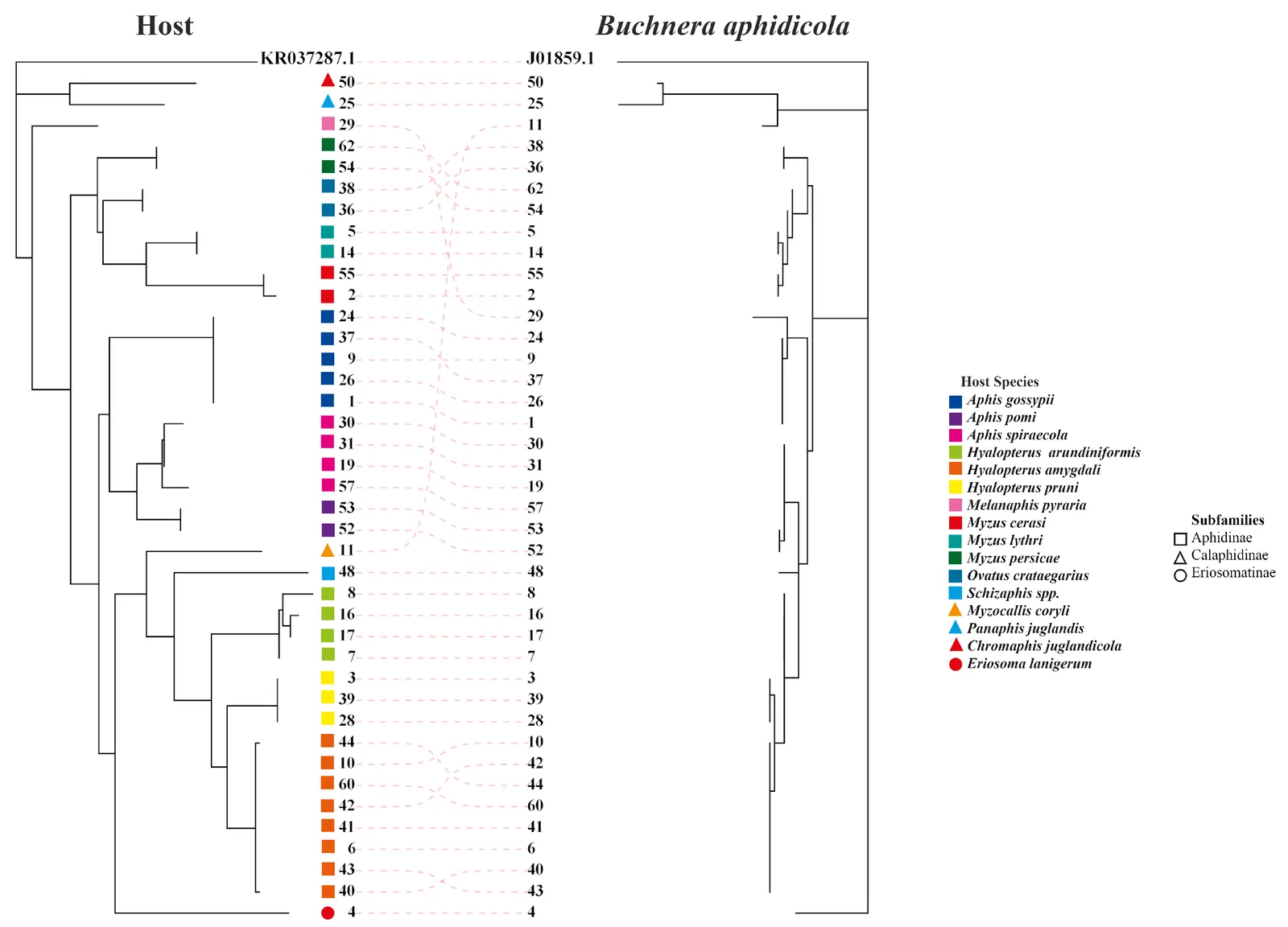
Tanglegram comparing the phylogenetic trees of aphid hosts (based on mitochondrial COI sequences, left) and their obligate endosymbiont *Buchnera aphidicola* (based on 16S rRNA sequences, right). Connecting dashed lines illustrate the topological congruence and incongruence between host and symbiont lineages. Numbered leaf nodes correspond to individual sample IDs specified in the text, with symbol shapes and colors representing host species and subfamilies as indicated in the legend.

The cophylogenetic relationship between aphid hosts and their associated *Buchnera aphidicola* lineages was evaluated using the Procrustean Approach to Cophylogeny (PACo). The analysis revealed significant global congruence between host and symbiont phylogenies (SS = 0.617, 9999 permutations, *p* < 0.0001), indicating that the observed host–symbiont associations were significantly more congruent than expected by chance. This significant congruence is consistent with a strong long-term host-associated evolutionary relationship between aphids and *Buchnera*, which is compatible with the predominantly vertical inheritance of *Buchnera*. The analysis showed that most host–symbiont associations contributed positively to the overall congruence, although several individual associations exhibited higher jackknife residual values, indicating localized phylogenetic mismatches. These deviations suggest that, while the general host–symbiont pattern is congruent, some specific associations do not fully follow the overall phylogenetic structure. Jackknife residual values for individual host–symbiont associations are provided in Supplementary Table S1. A qualitative comparison of aphid *COI* and *Buchnera 16S rRNA* phylogenies is shown in Figure 3. The tanglegram revealed a broadly congruent pattern between host and symbiont phylogenies, with several lineages showing correspondence between host clustering and *Buchnera* relationships. Consistent patterns were observed within *Hyalopterus* and *Aphis gossypii* lineages, where host groupings were largely associated with similar *Buchnera* clustering patterns. In contrast, several small sample lineages showed apparent topological mismatches, although these patterns should be interpreted cautiously given the limited replication. These observations represent qualitative comparisons of phylogenetic topology based on tanglegram visualization.

### 3.5. Overall Detection Patterns of Aphid-Associated Bacteria

**Figure 4:**
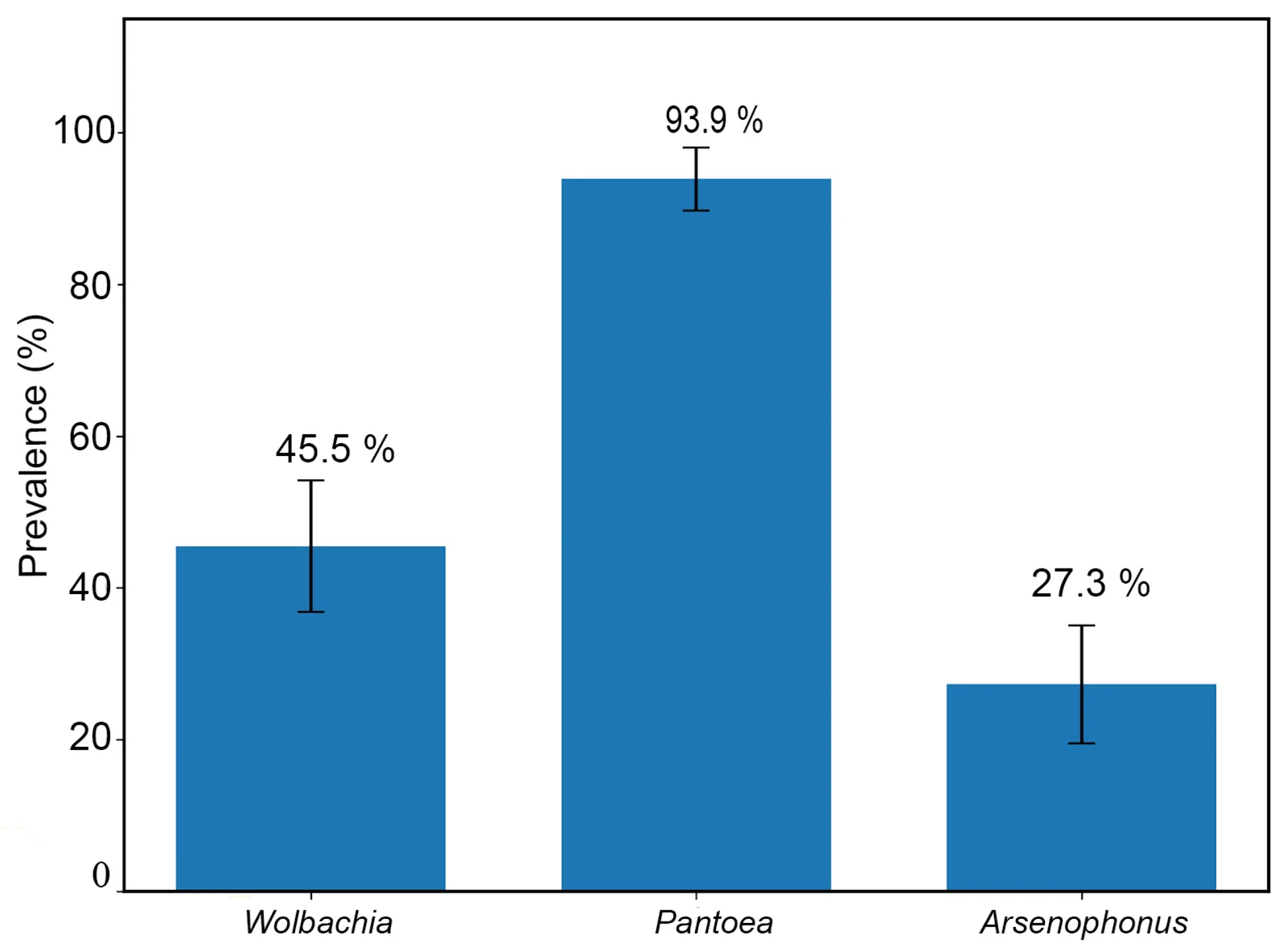
Overall detection patterns of aphid-associated bacteria in aphid samples. Bars represent the proportion of PCR-positive individuals (%, *n* = 33) for each bacterial taxon (*Wolbachia*, *Pantoea*, and *Arsenophonus*). Error bars indicate 95% exact binomial confidence intervals (Clopper–Pearson method).

The prevalence of aphid-associated bacteria is shown in Figure 4. Among the 33 screened aphid individuals, *Pantoea* was detected in 93.9% (31/33) of the samples (95% CI: 79.8–99.3), followed by *Wolbachia* (45.5%, 15/33; 95% CI: 28.1–63.6) and *Arsenophonus* (27.3%, 9/33; 95% CI: 13.3–45.5).

### 3.6. Species-Level Distribution Patterns of Detected Bacterial Taxa

**Figure 5:**
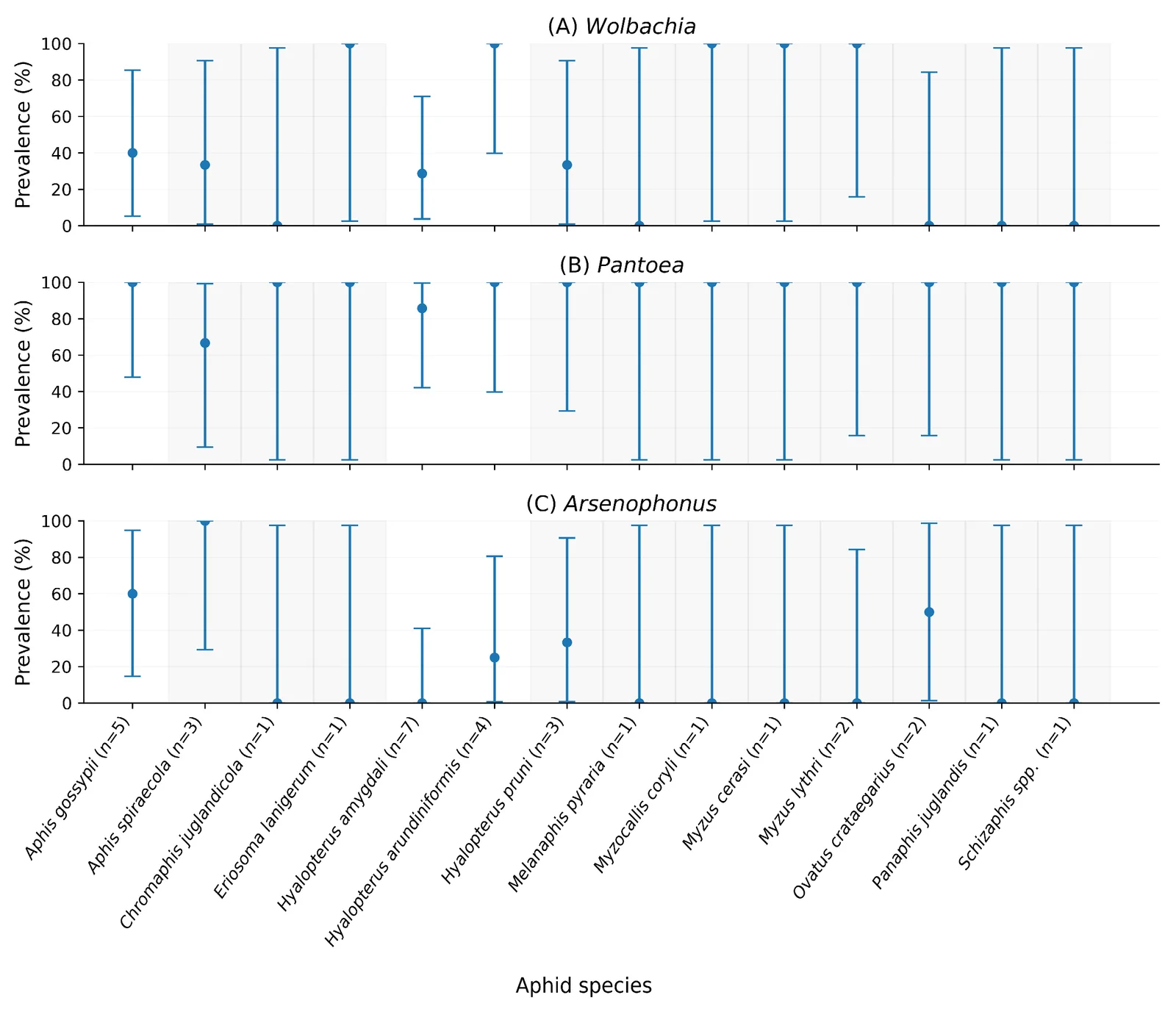
Species-level detection patterns of aphid-associated bacteria. Points indicate prevalence (%) for each aphid species; error bars represent 95% exact binomial confidence intervals (Clopper–Pearson method). Sample sizes (*n*) are shown for each species. Panels correspond to (**A**) *Wolbachia*, (**B**) *Pantoea*, and (**C**) *Arsenophonus*. Shaded areas denote species with *n* ≤ 3.

Species-level detection frequencies are shown in Figure 5. Given the uneven sample sizes among aphid species, these values should be interpreted as descriptive patterns rather than precise species-level prevalence estimates. *Wolbachia* showed variable detection patterns across aphid taxa, with detection observed in some species and not detected in others. *Pantoea* showed a broad distribution across the sampled host species, whereas *Arsenophonus* exhibited a more restricted and heterogeneous detection pattern. Species represented by small sample sizes (*n* ≤ 3) were interpreted cautiously because of the greater uncertainty associated with these observations.

### 3.7. Distribution Patterns of Detected Bacterial Taxa Across Aphid Species

**Figure 6:**
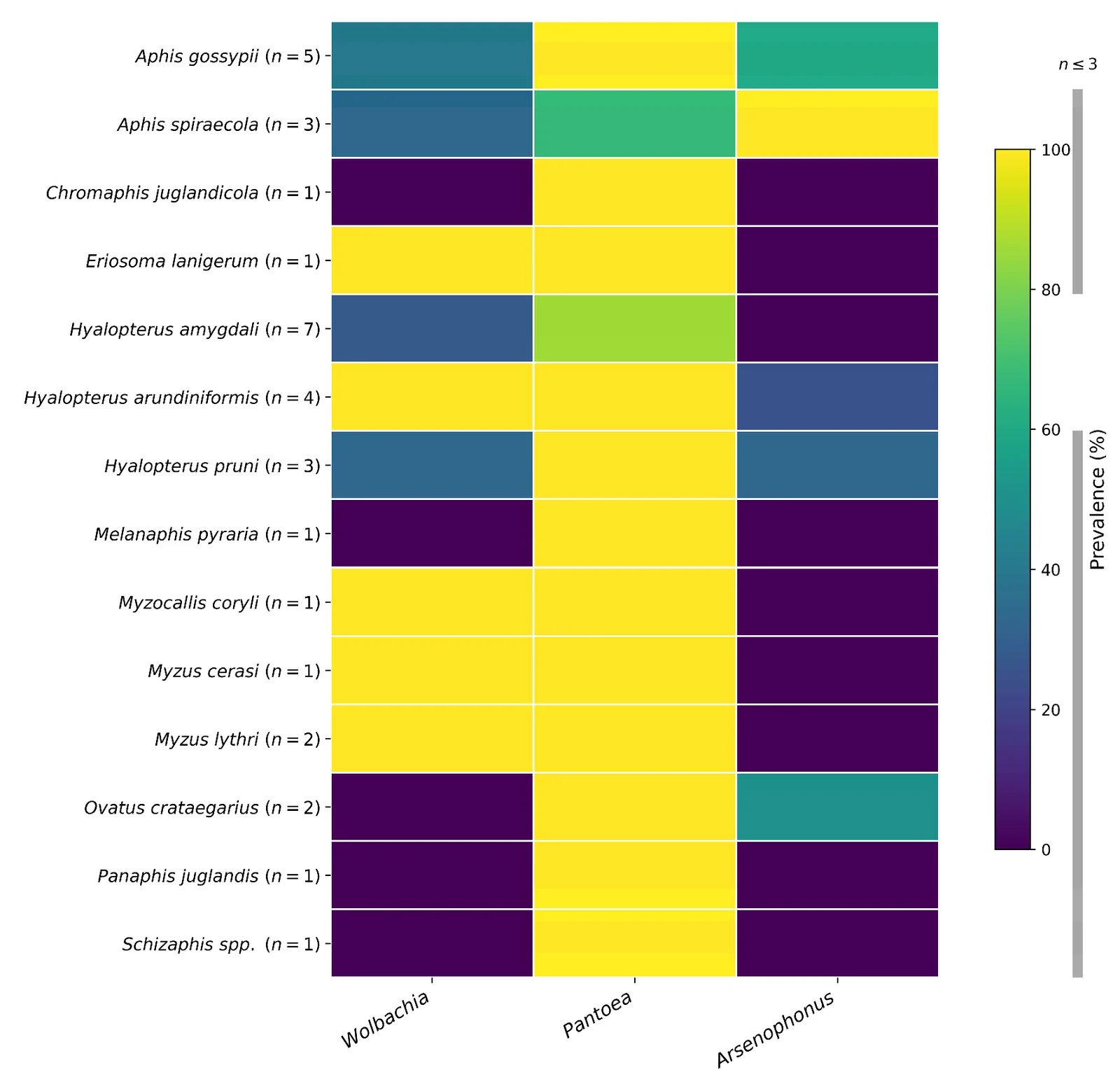
Distribution of aphid-associated bacteria across aphid species. Color intensity represents detection frequency (%) of each bacterial taxon within host species. Species with small sample sizes (*n* ≤ 3) are indicated with an asterisk and should be interpreted with caution.

The distribution patterns of detected aphid-associated bacteria taxa across host species are illustrated in Figure 6. The heatmap shows variation in bacterial detection patterns among aphid hosts. *Pantoea* was detected across most sampled host species, whereas *Wolbachia* showed a more variable detection pattern. *Arsenophonus* displayed a more restricted distribution, with detection limited to a subset of host taxa. Overall, these results indicate variation in the detection of aphid-associated bacteria across the sampled aphid hosts.

### 3.8. Pairwise Associations Among Detected Aphid-Associated Bacteria

**Figure 7:**
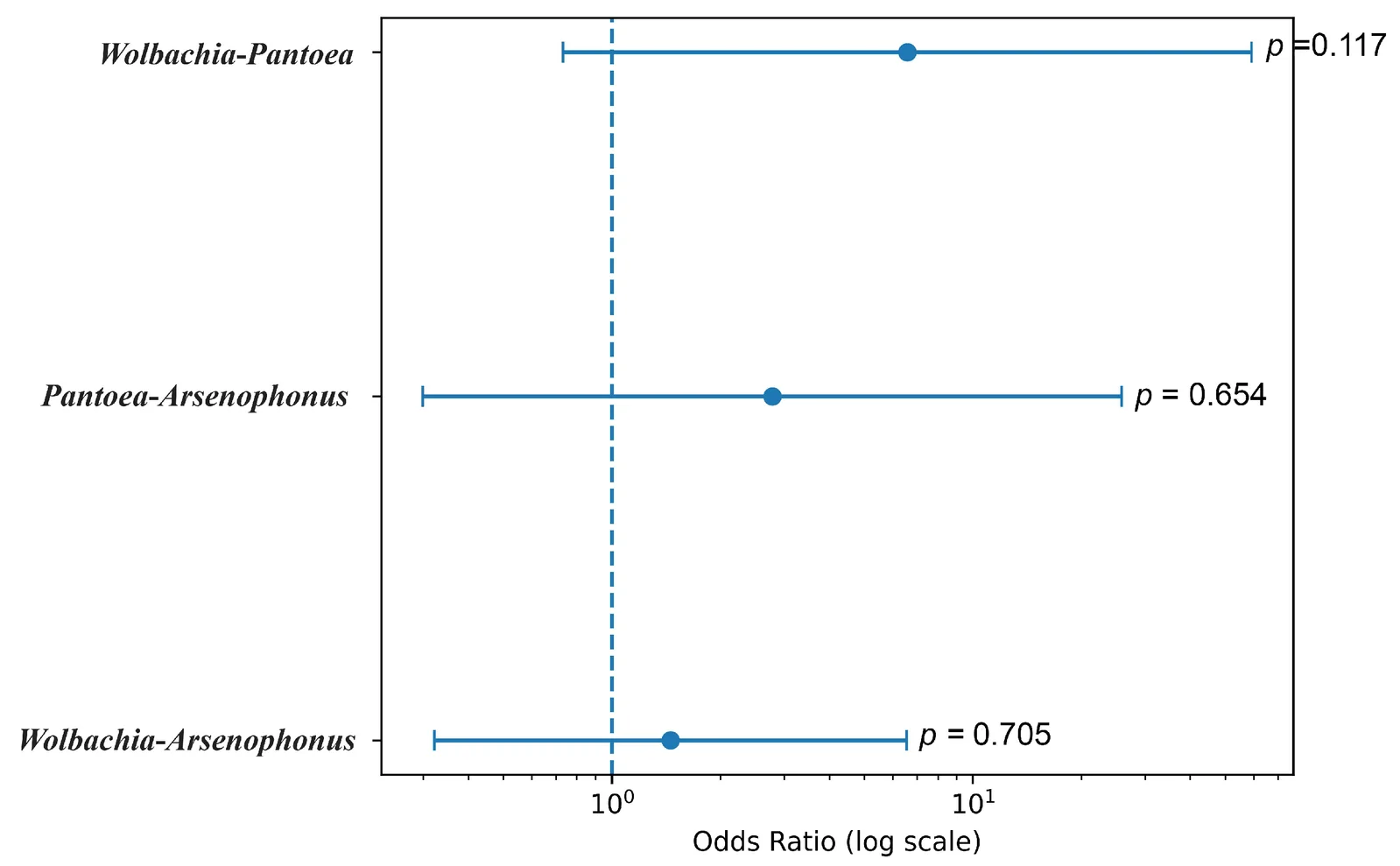
Pairwise associations among aphid-associated bacteria. Odds ratios (ORs) and 95% confidence intervals derived from Fisher’s exact tests are shown. The dashed vertical line indicates OR = 1. No statistically significant associations were detected (all *p* > 0.05).

Pairwise co-detection associations among aphid-associated bacteria are shown in Figure 7. Fisher’s exact tests did not detect statistically significant associations among the examined taxa (all *p* > 0.05). Estimated odds ratios were associated with wide confidence intervals overlapping the null expectation (OR = 1), indicating high uncertainty in the direction and strength of pairwise associations. The association between *Wolbachia* and *Pantoea* showed an odds ratio greater than one, suggesting a positive trend; however, this association was not statistically significant (*p* = 0.117). Similarly, no significant associations were detected between *Pantoea* and *Arsenophonus* (*p* = 0.654) or between *Wolbachia* and *Arsenophonus* (*p* = 0.705). Overall, no statistically supported pairwise codetection associations were detected among the examined aphid-associated bacteria.

## 4. Discussion

This study integrates host phylogenetic reconstruction with targeted screening of aphid-associated bacteria l taxa to examine patterns of bacterial detection across aphid lineages. The results reveal a clear contrast between the phylogenetically structured pattern observed for the obligate symbiont *Buchnera aphidicola* and the more heterogeneous detection patterns observed among other aphid-associated bacteria. The correspondence between host and *Buchnera* phylogenies indicates a strong long-term host-associated evolutionary relationship, which is compatible with predominantly vertical inheritance of *Buchnera*. In contrast, the variable detection patterns of other aphid-associated bacteria suggest that their distributions cannot be explained solely by host phylogenetic relationships and may also reflect ecological, environmental, or transmission-related processes. Given the small and uneven sample sizes among aphid species, these findings should be interpreted as descriptive screening patterns rather than species-level prevalence estimates. Rather than providing a comprehensive characterization of aphid-associated microbial communities, our targeted marker-based approach was designed to assess the occurrence and distribution of selected bacterial taxa across the sampled aphid hosts.

The composition of the sampled aphid community reflects ecological variation among aphid taxa, including species associated with Rosaceae hosts as well as taxa with relatively broad or restricted host associations. Such ecological differences may be relevant to bacterial acquisition and persistence, as host range and plant use have been suggested to influence aphid–bacteria associations [38,39]. The uneven sample sizes among species likely reflect differences in field occurrence and population abundance during sampling, but these differences also limit the extent to which species-level patterns can be compared. Therefore, the observed variation in bacterial detection should be interpreted primarily as descriptive screening patterns rather than evidence of host-species effects. This ecological heterogeneity provides important context for interpreting the distribution of aphid-associated bacteria across the sampled hosts.

Molecular analyses further supported the consistency of species identification, with COI-based phylogeny showing overall concordance with morphological assignments. This finding is consistent with the established utility of COI as a DNA barcode marker for aphids, particularly for species-level discrimination and shallow taxonomic comparisons [40,41]. However, the reduced resolution observed at deeper nodes reflects known limitations of mitochondrial markers for resolving higher-level evolutionary relationships, emphasizing the value of multilocus approaches in aphid phylogenetic studies [17,41]. Together, these results provide a useful host phylogenetic framework for interpreting the observed patterns of bacterial detection in an evolutionary context.

Similarly, the phylogeny of *Buchnera* revealed a general pattern of host-associated clustering, consistent with the expected influence of long-term host association on the evolutionary history of aphid obligate symbionts [10,42]. This pattern supports the view that obligate symbiont lineages are strongly structured by host evolutionary history rather than solely by short-term ecological processes. The overall correspondence between host and symbiont phylogenies was further supported by the cophylogenetic analysis, which revealed significant global congruence between aphid hosts and *Buchnera aphidicola* lineages. This significant congruence is consistent with a strong long-term host-associated evolutionary relationship, which is compatible with predominantly vertical inheritance of *Buchnera* but does not by itself demonstrate a particular transmission mode. However, the observed congruence was not complete, as several host–symbiont associations showed localized incongruences. These mismatches may reflect a combination of biological processes, such as differences in evolutionary rates or incomplete lineage sorting, as well as limitations associated with single-marker datasets. Therefore, the observed pattern supports broad host–symbiont correspondence rather than strict one to one cospeciation.

In contrast to the structured pattern observed for *Buchnera*, the aphid-associated bacteria l taxa examined here showed heterogeneous detection patterns across aphid host species. *Wolbachia* and *Arsenophonus* displayed considerable variation in detection frequencies among host taxa, whereas *Pantoea* showed a broader distribution across the analyzed aphid species. This pattern is consistent with previous studies indicating that aphid-associated bacteria l taxa can vary in distribution according to host identity, ecological conditions, and transmission dynamics [14,23]. Unlike obligate symbionts, some aphid-associated bacteria may be less tightly constrained by host phylogenetic history and may be influenced by processes such as horizontal transmission and ecological interactions. However, the present screening design does not allow the relative contributions of host identity, ecological conditions, or transmission processes to be independently evaluated. Therefore, the heterogeneous detection patterns observed in this study should be interpreted as descriptive patterns that may reflect multiple ecological and evolutionary processes rather than as evidence for specific transmission mechanisms.

A distinct pattern was observed for *Pantoea*, which was frequently detected across a broad range of aphid host taxa [43]. This widespread detection pattern differs from that commonly reported for several well-characterized facultative symbionts, which often show more restricted host distributions [14,44]. The high detection frequency of *Pantoea* may reflect environmental acquisition, transient association, or repeated exposure through host plant surfaces or shared environmental reservoirs. However, the present study was based on presence–absence PCR data without localization or quantitative measurements, and therefore, the biological nature, persistence, and mode of acquisition of the detected *Pantoea* cannot be conclusively determined. Accordingly, *Pantoea* is referred to here as an aphid-associated bacterium rather than a confirmed facultative endosymbiont. The observed pattern highlights that frequent PCR detection does not necessarily demonstrate a stable or functionally established symbiotic association and emphasizes the importance of distinguishing between stable host-associated bacteria and environmentally mediated bacterial associations in aphids.

Although differences in detection frequencies were observed among the screened bacterial taxa, pairwise analyses did not provide statistical support for nonrandom co-detection patterns among taxa. Given the wide confidence intervals and sparse contingency tables, these results should be interpreted cautiously and may partly reflect limited statistical power associated with the small sample size. Previous studies have shown that aphid-associated bacterial communities can involve complex ecological relationships, including potential synergistic and antagonistic interactions among bacterial taxa [14,23]. Therefore, the absence of statistically supported codetection patterns in the present dataset should not be interpreted as evidence that biological interactions are absent. Instead, potential associations may depend on ecological context, spatial variation, host background, or environmental conditions that were not independently evaluated in this study. Given the descriptive nature of the screening and the uneven sampling among aphid species, further studies with larger and independently replicated samples will be necessary to evaluate these potential associations more robustly.

Several limitations should be considered when interpreting these findings. First, the dataset was based on a relatively small number of individuals, and species-level representation was uneven, limiting the robustness of interspecific comparisons. Accordingly, the observed species-level patterns should be considered descriptive rather than as estimates of true prevalence. Second, bacterial screening was restricted to a targeted subset of aphid-associated taxa and therefore does not capture the complete diversity of bacteria associated with aphids. Third, the sampling design was not independently replicated across aphid species, host plants, geographic locations, or environmental conditions; therefore, the present dataset does not permit independent evaluation of host species, host plant, geographic, or environmental effects on bacterial detection patterns. Fourth, host–symbiont evolutionary comparisons were based on partial 16S rRNA data for *Buchnera*, which may limit phylogenetic resolution and the interpretation of deeper evolutionary relationships. Finally, reliance on PCR-based presence/absence data, particularly for broadly detected taxa such as *Pantoea*, limits the ability to distinguish between stable host-associated relationships, transient occurrence, and environmentally acquired bacterial associations.

Taken together, these findings indicate that obligate and other aphid-associated bacteria exhibit contrasting patterns of evolutionary and ecological association. The strong host-associated pattern observed for *Buchnera* contrasts with the more variable detection patterns of other aphid-associated bacterial taxa, suggesting that their distributions cannot be explained solely by host phylogenetic relationships. These patterns may also reflect ecological, environmental, or transmission-related processes, although the present study does not allow these factors to be evaluated independently. Future studies incorporating broader geographic sampling, larger and more balanced sample sizes, quantitative approaches, and localization-based methods will help clarify the processes underlying these bacterial associations and determine the functional significance of frequently detected bacterial taxa in aphid biology.

## Figures and Tables

**Table 1:** Collection details of aphid specimens used in this study, including aphid species identification, host plant species, sampling locations, collection dates, geographic coordinates, and number of individuals analyzed.

Specimen ID	Host Plant	Location	Coordinates (N-E)	Aphid Species	GenBank Accession [COI]	GenBank Accession [Buchnera 16S rRNA]
1	*Prunus persica* (L.) BATSCH.	Türkiye, Ankara, Beypazarı, Başören	40°01′60″ N, 32°13′32″ E	*Aphis gossypii*	PZ830616	PZ836570
9	*Punica granatum* L.	Türkiye, Ankara, Çubuk, Dedeler	40°17′17″ N, 33°09′49″ E	*Aphis gossypii*	PZ830618	PZ836574
24	*Prunus armeniaca* L.	Türkiye, Ankara, Beypazarı, İnözü	40°11′38″ N, 31°54′11″ E	*Aphis gossypii*	PZ830617	PZ836571
26	*Pyrus communis* L.	Türkiye, Ankara, Kahramankazan, Aşağıkaraören	40°17′06″ N, 32°44′54″ E	*Aphis gossypii*	PZ830619	PZ836572
37	*Punica granatum* L.	Türkiye, Ankara, Ayaş, Ortabereket	40°08′04″ N 32°25′32″ E	*Aphis gossypii*	PZ830620	PZ836573
52	*Malus domestica* (Suckow) Borkh.	Türkiye, Ankara, Kahramankazan, Aşağıkaraören	40°17′06″ N 32°44′54″ E	*Aphis pomi*	PZ830651	PZ836568
53	*Malus domestica* (Suckow) Borkh.	Türkiye, Ankara, Yenimahalle, Tarım Kampüsü	39°57′16″ N 32°48′35″ E	*Aphis pomi*	PZ830652	PZ836569
19	*Malus domestica* (Suckow) Borkh.	Türkiye, Ankara, Çubuk, Dedeler	40°17′17″ N 33°09′49″ E	*Aphis spiraecola*	PZ830644	PZ836575
30	*Malus domestica* (Suckow) Borkh.	Türkiye, Ankara, Beypazarı, İnözü	40°11′38″ N 31°54′11″ E	*Aphis spiraecola*	PZ830641	PZ836576
31	*Pyrus communis* L.	Türkiye, Ankara, Beypazarı, Uşakgöl	40°17′26″ N 31°55′28″ E	*Aphis spiraecola*	PZ830642	PZ836577
57	*Cydonia oblonga* L.	Türkiye, Beypazarı, Beypazarı, İnözü	40°11′38″ N 31°54′11″ E	*Aphis spiraecola*	PZ830643	PZ836578
50	*Juglans regia* L.	Türkiye, Ankara, Kahramankazan, Soğulcak	40°12′45″ N 32°46′18″ E	*Chromaphis juglandicola*	PZ830654	PZ836592
4	*Malus domestica* (Suckow) Borkh.	Türkiye, Ankara, Ayaş, Ortabereket	40°08′04″ N 32°25′32″ E	*Eriosoma lanigerum*	PZ830626	PZ836584
7	*Prunus persica* L.	Türkiye, Ankara, Çamlıdere, Alakoç	40°33′32″ N 32°28′39″ E	*Hyalopterus arundiniformis*	PZ830629	PZ836564
8	*Prunus armeniaca* L.	Türkiye, Ankara, Akyurt, Merkez	40°06′43″ N 33°08′14″ E	*Hyalopterus arundiniformis*	PZ830630	PZ836565
16	*Prunus armeniaca* L.	Türkiye, Ankara, Kızılcahamam, Yukarıçanlı	40°39′24″ N 32°41′47″ E	*Hyalopterus arundiniformis*	PZ830631	PZ836566
17	*Prunus persica* L.	Türkiye, Ankara, Beypazarı, Haydarlar	40°16′39″ N 31°56′06″ E	*Hyalopterus arundiniformis*	PZ830632	PZ836567
6	*Prunus cerasifera* L.	Türkiye, Ankara, Kalecik, Baykuşboğazı	40°08′47″ N 33°20′40″ E	*Hyalopterus amygdali*	PZ830633	PZ836553
10	*Populus nigra* L.	Türkiye, Ankara, Bala, Merkez	39°33′14″ N 33°07′16″ E	*Hyalopterus amygdali*	PZ830634	PZ836554
40	*Prunus amygdalus* L.	Türkiye, Ankara, Çubuk, Merkez	40°14′20″ N 33°01′44″ E	*Hyalopterus amygdali*	PZ830635	PZ836555
41	*Prunus armeniaca* L.	Türkiye, Ankara, Çubuk, Aşağıçavundur	40°15′47″ N 33°01′03″ E	*Hyalopterus amygdali*	PZ830636	PZ836556
42	*Prunus amygdalus* L.	Türkiye, Ankara, Kahramankazan, İçören	40°13′48″ N 32°44′19″ E	*Hyalopterus amygdali*	PZ830637	PZ836557
43	*Prunus amygdalus* L.	Türkiye, Ankara, Elmadağ, Lalahan	39°58′23″ N 33°07′08″ E	*Hyalopterus amygdali*	PZ830638	PZ836558
44	*Prunus armeniaca* L.	Türkiye, Ankara, Şereflikoçhisar, Merkez	38°56′40″ N 33°32′31″ E	*Hyalopterus amygdali*	PZ830639	PZ836559
60	*Prunus amygdalus* L.	Türkiye, Ankara, Çankaya, Merkez	39°53′26″ N 32°51′42″ E	*Hyalopterus amygdali*	PZ830640	PZ836560
3	*Pyrus communis* L.	Türkiye, Ankara, Ayaş, Ortabereket	40°08′04″ N 32°25′32″ E	*Hyalopterus pruni*	PZ830623	PZ836562
28	*Prunus domestica* L.	Türkiye, Ankara, Çubuk, Dedeler	40°17′17″ N, 33°09′49″ E	*Hyalopterus pruni*	PZ830624	PZ836561
39	*Prunus domestica* L.	Türkiye, Ankara, Akyurt, Merkez	40°06′43″ N 33°08′14″ E	*Hyalopterus pruni*	PZ830625	PZ836563
29	*Pyrus communis* L.	Türkiye, Ankara, Yenimahalle, Tarım Kampüsü	39°57′16″ N 32°48′35″ E	*Melanaphis pyraria*	PZ830646	PZ836581
11	*Corylus avellana* L.	Türkiye, Ankara, Kahramankazan, Soğulcak	40°12′45″ N 32°46′18″ E	*Myzocallis coryli*	PZ830655	PZ836590
2	*Prunus cerasus* L.	Türkiye, Ankara, Polatlı, Üçpınar	39°34′51″ N 32°04′58″ E	*Myzus cerasi*	PZ830622	PZ836579
55	*Prunus cerasus* L.	Türkiye, Ankara, Ayaş, Ilıca	40°03′46″ N 32°15′31″ E	*Myzus cerasi*	PZ830621	PZ836588
5	*Prunus amygdalus* L.	Türkiye, Ankara, Kızılcahamam, Çeltikçi	40°19′22″ N 32°27′20″ E	*Myzus lyithri*	PZ830627	PZ836586
14	*Prunus cerasus* L.	Türkiye, Ankara, Nallıhan, Davutoğlan	40°07′00″ N 31°38′00″ E	*Myzus lythri*	PZ830628	PZ836580
54	*Malus domestica* (Suckow) Borkh.	Türkiye, Ankara, Nallıhan, Yenice	40°04′11″ N 30°51′30″ E	*Myzus persicae*	PZ830648	PZ836587
62	*Prunus persica* L.	Türkiye, Ankara, Çankaya, Merkez	39°53′26″ N 32°51′42″ E	*Myzus persicae*	PZ830649	PZ836589
36	*Cydonia oblonga* L.	Türkiye, Ankara, Çamlıdere, Çamkoru	40°34′43″ N 32°31′39″ E	*Ovatus crataegarius*	PZ830645	PZ836582
38	*Cydonia oblonga* L.	Türkiye, Ankara, Yenimahalle, Tarım Kampüsü	39°57′16″ N 32°48′35″ E	*Ovatus crataegarius*	PZ830647	PZ836583
25	*Juglans regia* L.	Türkiye, Ankara, Yenimahalle, Çayyolu	39°53′04″ N 32°41′55″ E	*Panaphis juglandis*	PZ830653	PZ836591
48	*Pyrus communis* L.	Türkiye, Ankara, Yenimahalle, Atatürk Orman Çiftliği	39°56′01″ N 32°48′27″ E	*Schizaphis* spp.	PZ830650	PZ836585

**Table 2:** Primer sequences used for detection of aphid-associated bacteria.

Target Gene	Symbiont	Primer Name	Primer Sequence (5–3′)	Tm (°C)	Reference
*23S rDNA*	*Arsenophonus*	*Ars23S-1*	CGTTTGATGAATTCATAGTCAAA	50	[35]
		*Ars23S-2*	GGTCCTCCAGTTAGTGTTACCCAAC		
*wsp* gene	*Wolbachia*	*wsp81F*	TGGTCCAATAAGTGATGAAGAAAC	55	[36]
		*wsp691R*	AAAAATTAAACGCTACTCCA		
*16S rRNA*	*Pantoea*	*Pantoea-F*	ACGGAGGGTGCAAGCGTTAAT	56	[37]
		*Pantoea-R*	AGGTAAGGTTCTTCGCGTTGCA		

## Data Availability

The data supporting the findings of this study are available from the corresponding author upon reasonable request.

## Supplementary Materials

The following supporting information can be downloaded at: https://www.mdpi.com/article/10.3390/insects17080875/s1, Table S1: Jackknife residuals for each aphid host-Buchnera aphidicola association obtained from the Procrustean Approach to Cophylogeny (PACo) analysis. Lower residual values indicate stronger phylogenetic congruence between host and bacterial lineages.
